# Preparation, characterization and activity verification of xylooligosaccharides from plantain straw

**DOI:** 10.1016/j.heliyon.2024.e40905

**Published:** 2024-12-04

**Authors:** Wenming Jiang, Fang Li

**Affiliations:** a School of Environmental and Quality Testing, Chongqing Chemical Industry Vocational College, Chongqing, 401228, China; b Chongqing Jiangbei District Disease Control Center, Chongqing, 400020, China; c Chongqing (Changshou) Industrial Technology Research Institute of Green Chemical and New Material, Chongqing, 401228, China

**Keywords:** Plantain straw, Xylooligosaccharides preparation, Structural characterization, Antioxidant activity

## Abstract

Currently, the utilization value of plantain straw is low. To increase its value, plantain straw was utilized in this study to produce xylooligosaccharides (XOS). XOS were obtained from plantain straw through xylanase hydrolysis. The antioxidant and probiotic properties of XOS were also investigated. UV–Visible scanning showed no notable absorption peaks at 260 and 280 nm, indicating that the protein and nucleic acid in hemicellulose B were removed by the Sevag method. High–performance liquid chromatography revealed that hemicellulose B consists of L–guluronic acid, D–mannuronic acid, D–mannose, D–glucosamine, L–rhamnose, D–glucuronic acid, D–galacturonic acid, D–galactosamine, D–glucose, D–galactose, D–xylose, L–arabinose, and L–fucose. Xylose, galacturonic acid, arabinose, glucose, and rhamnose accounted for 35.04 %, 14.50 %, 13.63 %, 11.87 %, and 10.29 %, respectively. Scanning electron microscopy revealed that XOS displayed cross–linking and a rough surface. Fourier transform infrared spectroscopy analysis indicated that the oligosaccharides contained β–glycosidic bonds. Nuclear magnetic resonance analysis revealed that XOS contain methyl and methoxy groups and are linked by 1–4 glycosidic bonds. In addition, compared to hemicellulose B, XOS showed superior DPPH scavenging ability and higher reducibility. Antimicrobial activity analysis demonstrated that XOS from plantain straw exhibited beneficial probiotic effects on *Lactobacillus acidophilus* and *Bifidobacterium animalis*.

## 1 Introduction

Musa basjoo Siebold & Zucc. ex Iinuma, native to the Ryukyu Islands, is a perennial herb belonging to the genus Musa in the Musaceae family. Musa basjoo is rich in nutrients and possess significant medicinal value. Therefore, it is cultivated in southern China, as well as in some areas of Shaanxi, Gansu, and Henan. To ensure a fruitful second year, plantain trees need to be pruned in winter to minimize nutrient wastage. As a result, a large amount of plantain stalk is produced every year. Plantain contains rich saccharides.

Carbohydrates can be used as auxiliary materials in the design of multiphase nanomaterials and have a promising application outlook in various fields. Glucose, fructose, lactose, maltose, and cellulose can serve as auxiliary components in TmVO_4_ nanostructures [1]. Starch polysaccharides play a crucial role in constructing carbon–based nanomaterials [2]. Saccharides can be utilized as fuel for the Pechini method to fabricate nanomaterials [3,4]. For instance, spherical YCMO nanoparticles were synthesized using the sol–gel self–combustion method with grape juice as a fuel and complexing agent, demonstrating significant potential in hydrogen storage [5]. Furthermore, nanocomposites based on chitosan can exhibit enhanced photocatalytic performance [6].

In particularly, plantain straw is particularly rich in organic matter such as cellulose, which offers a wide range of potential for comprehensive utilization. Firstly, plantain straw's abundance of cellulose makes it an ideal raw material for cellulose production [7]. Cellulose is a crucial biomass material that can be used in the manufacturing of paper, fiberboard, and bioplastics. Secondly, plantain straw can enhance soil quality [8]. With its high organic matter content, plantain straw can serve as a soil conditioner to improve soil structure and fertility. By reintroducing plantain straw to the soil, the organic matter content can be increased, leading to improved soil water retention and fertilizer conservation. Thirdly, plantain straw can also function as a renewable raw material for bioenergy. By utilizing biomass energy technologies, it can be transformed into bio–oil and biogas to substitute traditional fossil fuels [9]. Currently, there is limited research on plantain straw, and its application value is relatively low.

In addition to the aforementioned saccharides, plantain straw also contains a significant amount of hemicellulose, and its hydrolysis will yield more functional XOS. XOS, as a functional polysaccharide and dietary fiber, are widely utilized as additives [10]. Research has demonstrated that XOS can enhance animal growth performance, boost immunity, and stimulate the growth of bifidobacteria [11,12]. Moreover, XOS have been shown to have an inhibitory effect on *Staphylococcus aureus* [13] and can suppress inflammation induced by Salmonella [14]. Bifidobacterium exhibited vigorous growth and metabolic characteristics when exposed to XOS. Additionally, XOS were found to possess strong antioxidant properties, which can help regulate blood glucose levels and reduce blood lipids [15,16]. They can even be utilized as an anticancer ingredient [17]. Therefore, XOS hold significant research value.

However, so far, there has been no research on the XOS of plantain straw. In this study, XOS were first prepared from plantain straw, and their biological activities of plantain straw were investigated to provide insights for its further utilization. This not only effectively addresses the issue of agricultural waste treatment but also promotes the sustainable development of the agricultural industry.

## 2 Materials and methods

### 2.1 Materials

The plantain straw was collected from Changshou, Chongqing. Dialysis bags were purchased from Shanghai Yuanye Biotechnology Co., Ltd. Xylanases were obtained from Beijing Solaibao Technology Co., Ltd. Sodium hydroxide, ammonium oxalate, ethanol, glacial acetic acid, trichloroacetic acid, potassium ferricyanide, n–butanol, and 3,5–dinitrosalicylic acid were purchased from Shanghai McLean Biochemical Technology Co., Ltd. DPPH was acquired from Jinkong (Beijing) Biotechnology Co., Ltd. Chloroform and sulfuric acid were obtained from Bide Pharmaceutical (Shanghai, China). *Lactobacillus acidophilus* and *Bifidobacterium animalis* were isolated from yogurt.

### 2.2 Extraction of hemicellulose B

Plantain straw was crushed after drying, and then extracted in a boiling water bath with a 0.2–0.3 % sodium hydroxide solution for 1 h. The filtered residue was extracted with 0.8 % ammonium oxalate in a boiling water bath for 1 h, and dietary fiber was obtained through filtration. Dietary fiber was extracted using a 1–2% sodium hydroxide solution in a 40 °C water bath for 12–24 h. The filtered solution obtained through filtration was adjusted to a pH of approximately 5 using glacial acetic acid. The neutralization solution was centrifuged at 5000 rpm for 5 min to remove the precipitation. Hemicellulose B was obtained by centrifugation after adding three times the volume of absolute ethanol to the supernatant [18]. Hemicellulose B protein was removed using the Sevag method. The polysaccharide was dialyzed using a 3500 Da dialysis bag. The dialysate was freeze–dried and then stored for later use.

### 2.3 Protein and nucleic acid detection

Plantain hemicellulose B was dissolved in distilled water to achieve a concentration of 1–2 mg/ml. Insoluble substances were removed by centrifugation. The absorbance of the solution in the wavelength range of 200–600 nm was measured using an ultraviolet–visible spectrophotometer (Shenzhen Keliyixiang instrument equipment co., ltd; UV–1800) with distilled water as the control [19].

### 2.4 Monosaccharide detection

A 10–20 mg polysaccharide sample was dissolved in 5 ml of 2 mol/L trifluoroacetic acid solution. Under nitrogen protection, the solution was hydrolyzed in an oven at 110 °C for 2 h. After the hydrolysate cooled, 1 mL was taken out and mixed with 1 mL of methanol. The mixture was dried with N_2_ in a 70 °C water bath to eliminate trifluoroacetic acid. Subsequently, 1 mL of 0.3 mol/L NaOH solution was added to fully dissolve the hydrolyzed dry product.

The monosaccharide alkali solution was added to a 5 mL tube with a stopper, followed by adding 400 μL of 0.5 mol/L 1–phenyl–3–methyl–5–pyrazolone in methanol. The mixed solution was reacted in a 70 °C water bath for 2 h. After reaching room temperature, 0.3 mol/L hydrochloric acid solution was added to the reaction mixture to adjust the pH to 6–7. Subsequently, 1.2 mL of water and an equal volume of chloroform were added to the neutralized solution. The mixed solution was shaken, and the chloroform was removed. The filtered aqueous phase was passed through a 0.45 μm microporous membrane for High–performance liquid chromatography (HPLC) analysis.

The Agilent 1100 (DAD detector) is used to detect monosaccharide composition. Before using the HPLC instrument, a series of inspections were carried out to ensure the accuracy of the analysis and the safe operation of the instrument. Chromatographic conditions are as follows: C18 column (250 mm × 4.6 mm); Mobile phase A: 100 mM sodium phosphate buffer (pH = 6.7); Mobile phase B: acetonitrile; Detection wavelength: 250 nm; Column temperature: 30 °C; Flow rate: 1 mL/min; Injection volume: 5 μL. The gradient elution conditions were as follows: 0 min phase A/phase B (86:14, v/v), 9 min phase A/phase B (83:17, v/v), 28 min phase A/phase B (78:22, v/v), 29 min phase A/phase B (50:50, v/v), 32 min phase A/phase B (86:14, v/v).

### 2.5 Preparation of XOS

Hemicellulose B (0.5 g) was dissolved in 20 mL of buffer solution with a pH of 4.5. Xylanase was added to the solution to achieve a concentration of 200 U/g substrate. The solution was hydrolyzed at 45 °C for 1, 2, 3, 4, 5, 6, 7, 8 and 9 h to determine the degree of hydrolysis at different times. The hydrolysate was centrifuged after being placed in a boiling water bath to remove precipitation. The concentration of reducing sugar in the supernatant was determined using 3,5–Dinitrosalicylic acid (DNS). Sulfuric acid was added to the supernatant to increase its concentration to 6 %. After 2 h of boiling the hydrolysate in a water bath, the DNS method was also employed to determine the total sugar content and calculate the degree of polymerization [20].

Activated carbon particles were activated and filled into a 22 mm × 400 mm chromatography column. The enzymatic hydrolysate was loaded onto the activated carbon in the chromatographic column at a consistent rate. The chromatographic column was washed with 300 mL of deionized water and eluted with a 60 % ethanol solution. The flow rates mentioned above were all set at 1 mL/min. After the ethanol eluent was removed by rotary evaporation, the solution was freeze–dried for standby.

### 2.6 Characterization of oligosaccharides

Dried XOS sample of 0.005 g was mixed with 0.5 g of KBr powder in an agate mortar and ground. Using KBr as the background, an FTIR–650 Fourier transform infrared spectrometer (Shanghai Leao Test Instrument Co., Ltd.) was used to measure the transmissivity in the wave number range of 4000–450 cm^−1^ [21].

A dry sample weighing 5 mg was attached to the conductive carbon film using double–sided adhesive. The conductive carbon film was then positioned in the sample chamber of the ion sputtering equipment and sprayed with gold for approximately 40 s. Subsequently, the sample was moved to the observation room of a scanning electron microscope (Zeiss Sigma 500) and observed at an accelerating voltage of 2 kV [22]. Before the use of Scanning electron microscopy (SEM), a series of inspections were carried out to ensure the normal operation of the equipment and obtain high–quality images.

The 25 mg sample was dissolved in D_2_O, and the spectrum of the plantain stalk XOS sample was recorded using a 500 MHz Nuclear magnetic resonance (NMR) spectrometer (Bruker) at 25 °C [23].

### 2.7 Determination of antioxidant activity *in vitro*

A 1 mL sample solution and pretreated DPPH ethanol solution (with a mass fraction of 0.004 %) of 4 ml were thoroughly mixed in a test tube. The reaction took place at room temperature in the dark for 30 min. The absorbance was measured at 517 nm, and the test was repeated three times for each group. The DPPH clearance rate was calculated as shown in Formula 1, where: A_0_ is the absorbance value of the DPPH ethanol solution and ethanol sample of the same volume; A_1_ is the absorbance value of the DPPH ethanol solution and sample; A_2_ is the absorbance value of the ethanol solution of the sample without DPPH.

Formula 1 $$\text{DPPH}\;\text{radical}\;\text{scavengin}g\;\text{rate}\;\left(\%\right)=\frac{A_{0}-\left(A_{1}-A_{2}\right)}{A_{0}}\times 100$$

After shaking and mixing 1 mL of the sample solution, 2.5 mL of 0.2 mol/L phosphate buffer solution (pH 6.6), and 2.5 mL of 1 % potassium ferricyanide solution, the mixture was immersed in water at 50 °C for 20 min. After cooling to room temperature, 2.5 mL of trichloroacetic acid solution with a mass fraction of 10 %, 0.4 mL of ferric chloride solution with a mass fraction of 1 %, and 4 mL of distilled water were added to the mixture. The reaction was then allowed to stand for 15–20 min. The reaction solution was centrifuged at 3500 rpm for 5 min. The absorbance of the supernatant was measured at 700 nm. The test was repeated three times in each group. The reducibility was based on Formula 2, where: A_0_ is the absorbance value of the blank group solution; A_1_ is the absorbance value of the sample group; A_2_ is the absorbance value of the control group.

Formula 2 $$\text{Reducing}\;\text{powe}r\;\left(\%\right)=\left(1-\frac{A_{1}-A_{0}}{{A\;}_{2}}\right)\times 100$$

### 2.8 Verification of bacterial activity

*Lactobacillus acidophilus* and *Bifidobacterium animalis* were cultured in MRS liquid medium at 37 °C for 48 h to activate them. Subsequently, they underwent two additional rounds of reactivation using the same procedure. MRS liquid medium without glucose was prepared and adjusted to pH 6.8. Two 5 mL MRS media were added with 5 mg of glucose and XOS, respectively. After sterilization at 121 °C for 15 min, 4 % (v/v) activated bacteria were inoculated. The bacterial solution was incubated in oscillatory anaerobic culture at 37 °C, and the absorbance at 600 nm was measured every 4 h [24].

### 2.9 Data analysis

All data are expressed as the mean ± standard deviation. The experiment was repeated three times. ANOVA was used to analyze the data results, and the difference was considered significant when p < 0.05.

## 3 Results and discussion

### 3.1 Detection of polysaccharide and monosaccharide composition

**Fig. 1:**
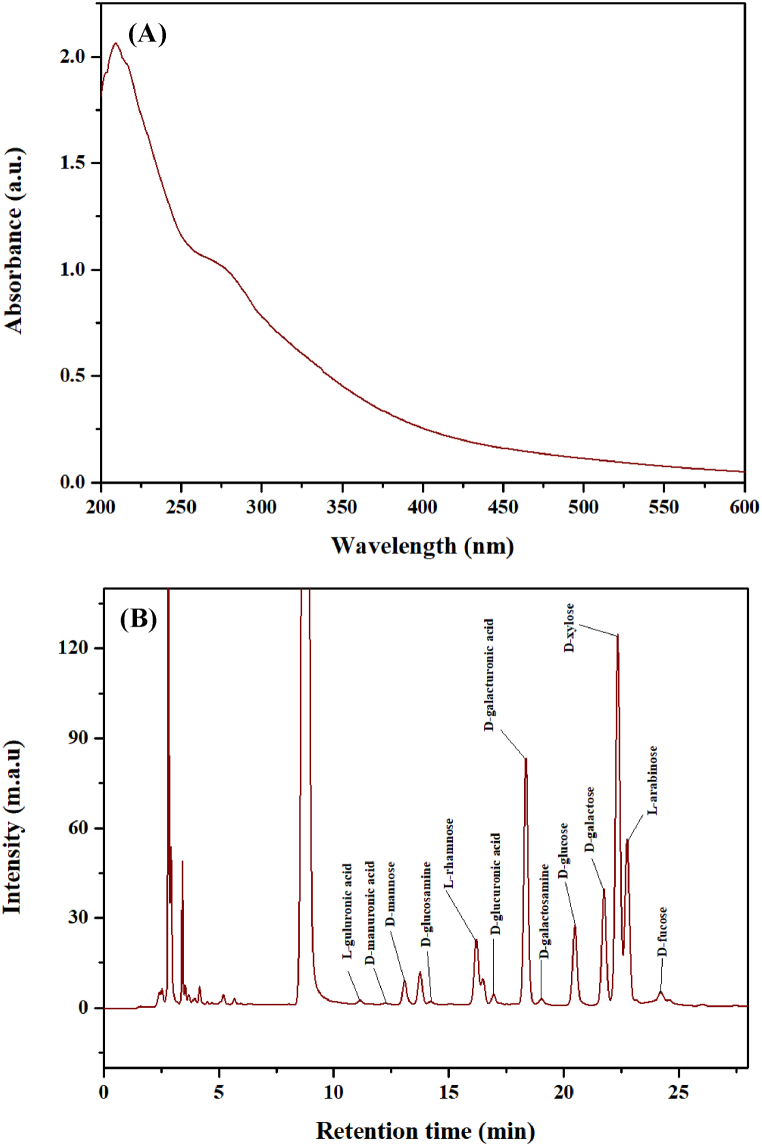
Polysaccharide analysis and monosaccharide composition of plantain stem (A) UV–Visible absorption spectrum and (B) HPLC chromogram of hemicellulose B.

To determine whether nucleic acids and proteins have been removed, the absorption at 260 and 280 nm was typically analyzed through UV–Vis scanning [25]. As shown in Fig. 1(A), the UV spectrum scanning of XOS from plantain straw reveals no distinct absorption peaks at 260 nm and 280 nm, indicating the successful removal of nucleic acids and proteins from the sample.

HPLC is an analytical technique widely utilized in chemical, food, and other industrial production. It often uses standards as controls to analyze monosaccharide composition [26]. In this study, the composition of plantain straw hemicellulose B was determined. The experimental results are shown in Fig. 1(B). Hemicellulose is composed of L–guluronic acid, D–mannuronic acid, D–mannose, D–glucosamine, L–rhamnose, D–glucuronic acid, D–galacturonic acid, D–galactosamine, D–glucose, D–galactose, D–xylose, L–arabinose, and L–fucose. Xylose is the most abundant component, accounting for 35.040 % of the total sugar content, followed by galacturonic acid and arabinose (Table 1).

**Table 1:** Monosaccharide composition of plantain stem hemicellulose B.

Sl. No.	Monosaccharide	Retention time (min)	Mass concentration (mg/g)	Mole percent (%)
1	L–guluronic acid	11.133	2.492	0.271
2	D–mannuronic acid	12.235	1.051	0.115
3	D–mannose	13.081	15.813	1.857
4	D–glucosamine	14.219	5.058	0.496
5	L– rhamnose	16.199	79.861	10.291
6	D– glucuronic acid	16.950	7.977	0.869
7	D–galacturonic acid	18.357	133.098	14.502
8	D–galactosamine	19.035	6.577	0.646
9	D–glucose	20.485	101.128	11.874
10	D–galactose	21.752	66.513	7.089
11	D–xylose	22.350	248.687	35.040
12	L– arabinose	22.758	96.705	13.626
13	L–fucose	24.222	20.218	2.605

### 3.2 Preparation and observation of XOS

**Fig. 2:**
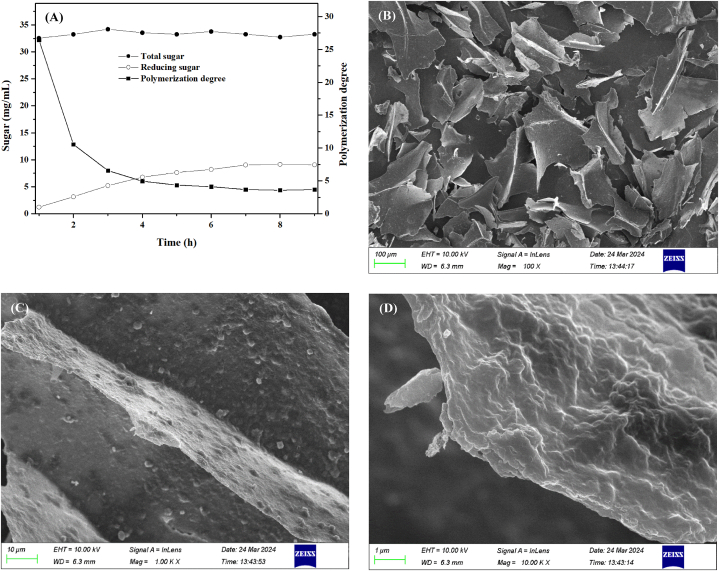
Enzymatic hydrolysis and SEM observation of hemicellulose B (A) Enzymatic hydrolysis of hemicellulose B; (B)–(D) SEM images with magnification of 100, 1000 and 10000 times.

The prolonged enzymatic hydrolysis will result in an increase in monosaccharide content and a decrease in XOS content. Insufficient enzymolysis time can result in a high degree of polymerization and inadequate enzymolysis. It can be seen from Fig. 2(A) that the total sugar content began to decrease slowly after 3 h of enzymatic hydrolysis. After 4 h of enzymatic hydrolysis, the degree of polymerization began to decrease slowly. The degree of polymerization was maintained at 4–5 after enzymatic hydrolysis for 4–6 h. After 6 h of enzymatic hydrolysis, the degree of polymerization remained at 3–4 and did not decrease significantly. The reducing sugar content decreased gradually after 4 h of enzymatic hydrolysis. The level of reducing sugar remained constant after 7 h of enzymatic hydrolysis. According to the reduction in sugar content and polymerization degree, the enzymolysis time was set at 4–7 h.

In this study, the surface structure of XOS from plantain straw was observed using SEM. Fig. 2(B)–(D) show the surface structure at 100x, 1000x, and 10000x magnifications, respectively. After magnification of 100 times, the oligosaccharides were crosslinked into an irregular fragmented structure [27]. When magnified at 1000 and 10000 times, the surface of XOS appeared rough and opaque.

### 3.3 FT–IR and one dimensional NMR analysis

**Fig. 3:**
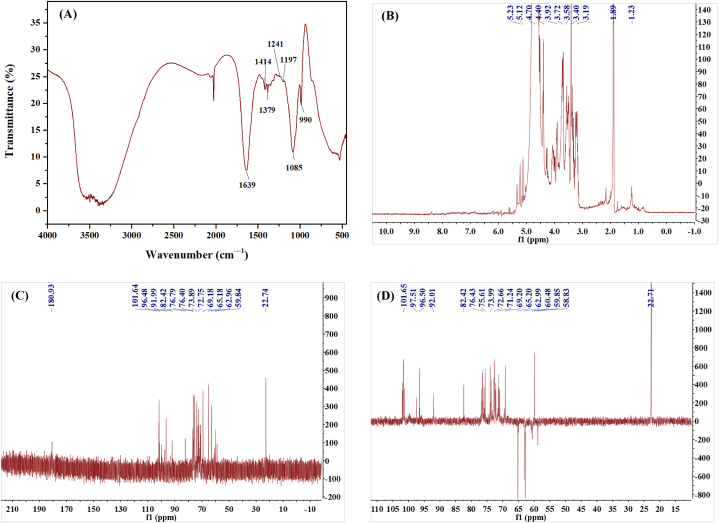
FT–IR and one–dimensional NMR analysis of XOS (A) FT–IR analysis of XOS; (B)–(C) ^1^H, ^13^C and DEPT135 observation of XOS.

Fourier transform infrared spectroscopy (FT–IR) is widely used to analyze and identify the chemical bonds and functional groups of molecules. The structure of oligosaccharides, including sugar configuration, glycosidic bond type, and functional groups, can be analyzed using FT–IR. It can be seen from Fig. 3(A) that the absorption peaks of oligosaccharides at 3500 cm^−1^ are disordered, including the tensile bending dynamic absorption peak of O–H, the bending vibration absorption peak of –COOH, and the absorption peak of N–H [28,29]. The absorption peak at 1633 cm^−1^ corresponds to the stretching vibration absorption of –OH [30]. The absorption peaks at 1414 cm^−1^ and 1379 cm^−1^ correspond to the bending deformation of –CH_2_– and the symmetrical deformation of –CH_3_, respectively [31]. The absorption peak at 1241 cm^−1^ is the C–H bending vibration absorption [32]. The absorption peak at 1197 cm^−1^ corresponds to the C–O absorption on the sugar ring [33,34]. The strong characteristic peak at 990 cm^−1^ is associated with the β–glycosidic bond [35].

NMR analysis of saccharides aims to determine the stereochemical configuration of polysaccharides and the linkage pattern of glycosidic bonds by recording the chemical shifts of hydrogen and carbon atoms under a high–frequency magnetic field. One–dimensional NMR includes ^1^H NMR, ^13^C NMR, and DEPT 135, which can be used to assign the chemical shifts of carbon and hydrogen in sugar residues and analyze the types of carbon atoms in oligosaccharides. In this study, one–dimensional NMR was used to characterize the structure of XOS from plantain straw. The results are shown in Fig. 3(B)–(D). As can be seen from the ^1^H NMR spectrum (Fig. 3(B)), the signals in the ^1^H NMR spectrum are concentrated in the range of 3.19–5.23 ppm, with a small portion distributed in the range of 1.23–2.19 ppm. In general, the chemical shift value of α–glycosyl isomers is more than 5.0 ppm, while the signal value of β–glycosyl isomers is less than 5.0 ppm [36]. The hydrogen spectrum signals of XOS were mainly concentrated in the range of less than 5 ppm, indicating that most of them were β–glycosidic bonds and contained a small amount of α–glycosidic bonds. The chemical shift range of 3.19–3.92 ppm is primarily associated with the hydrogen chemical shifts on carbons C2 to C6. Chemical shift of hydrogen on C1 corresponds to 4.40–5.23 ppm. In addition, the signal peaks at 1.23 ppm and 1.89 ppm are usually characteristic peaks of methyl [37].

The ^13^C NMR spectra of XOS are shown in Fig. 3(C). The chemical shift is 180.93 ppm, corresponding to the carbonyl group (–CO–) of an aldonic acid [38]. The chemical shift of C1 is 96.48–101.64 ppm. The absorption peak at 62.96–82.42 ppm corresponds to the chemical shift of C2–C6. The chemical shift of 22.74 ppm corresponds to the methyl signal peak [39], while the signal peak near 59.84 ppm is attributed to methoxy [40]. In DEPT 135, CH and CH_3_ exhibit positive signal peaks, whereas CH_2_ shows negative signal peaks [41]. According to the observation results of DEPT 135 (Fig. 3(C)), the signal is positive at 92.50–101.65 ppm, which is consistent with C1 in the sugar ring. The chemical shift of 69.20–76.43 ppm represents the positive signal peak, corresponding to C2–C4. The chemical shift of 62.85–65.20 ppm represents the negative signal peak, which is likely associated with C5 of pentose. The negative peaks at 60.48 and 58.83 ppm correspond to C6 of a hexose. The chemical shift of 59.85 ppm may correspond to the signal peak of –OCH_3_, while 22.71 ppm may represent the signal peak of methyl [38].

### 3.4 Two–dimensional NMR spectra

**Fig. 4:**
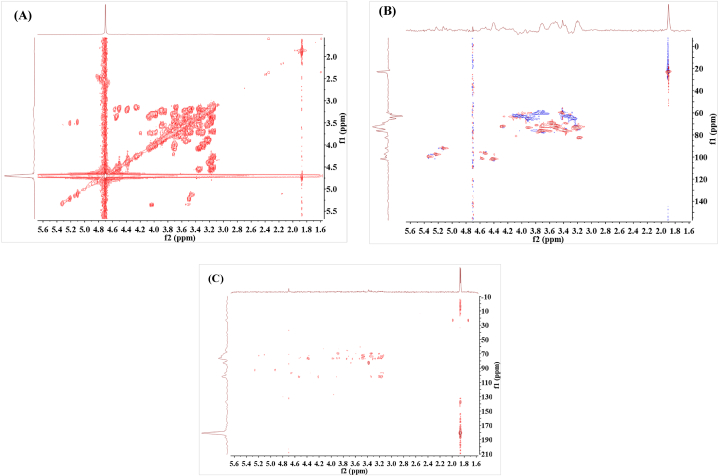
Two–dimensional NMR of XOS from soybean straw (A) COSY; (B) HSQC; (C) HMBC.

Two–dimensional NMR plays a crucial role in the analysis of molecular structures. To further analyze the molecular structure of XOS, two–dimensional NMR analysis was conducted. COSY is the most commonly used synuclear shift correlation spectrum, which reveals the coupling relationship between adjacent carbon and hydrogen atoms on the same carbon. Hydrogen atoms on the same carbon in the sugar molecule intersect on the diagonal [42]. As can be seen from Fig. 4(A), the ^1^H signal coupling of adjacent carbons is relatively strong. Additionally, it is relatively dense along the diagonal line, primarily due to the coupling of hydrogen on methyl, pentose C5, and hexose C6. Methyl and methoxy groups were found on the diagonal. The HSQC spectrum is a correlation spectrum between adjacent C–H atoms, which can directly associate the ^1^H nucleus with the ^13^C nucleus connected to it, thereby solving the connection relationship between C–H [43]. According to the HSQC spectral analysis (Fig. 4(B)), the methyl group is connected to C1 of the first sugar residue of XOS or C4 of the tail sugar residue, and the methoxy group is connected to C5. HMBC spectrum is a C–H heteronuclear multiple bond correlation spectrum, which can detect the coupling signal between hydrogen and remote carbon [44]. The linker fragments and sequences of monosaccharide residues can be inferred by coupling heterohead hydrogen with carbon attached to another sugar residue. It can be seen from Fig. 4(C) that sugar residues are connected by 1–4 glycosidic bonds.

### 3.5 Activity verification

**Fig. 5:**
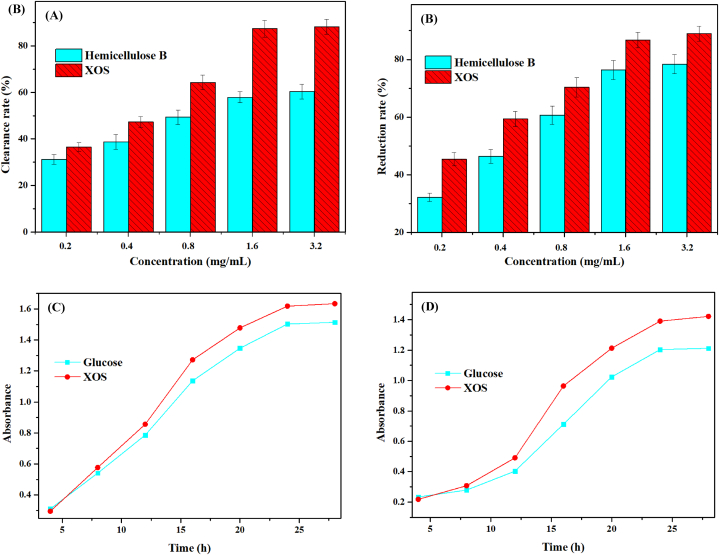
Antioxidant and probiotic activity (A) DPPH clearance of XOS; (B) Reducibility of XOS; (C) Probiotic activity of XOS to *Lactobacillus acidophilus*; (D) Probiotic activity of XOS to *Bifidobacterium animalis*.

Xylan is a type of heterogeneous polysaccharide found in plant cell walls, constituting 15 %–35 % of the dry weight of plant cells. It is the main component of plant hemicellulose B, and its monosaccharide composition includes pentose and hexose. XOS produced through its decomposition exhibit strong antioxidant and probiotic microbial activity. In this study, the antioxidant activity of XOS was evaluated using DPPH clearance and reduction ability, while the probiotic activity was assessed with *Lactobacillus acidophilus* and *Bifidobacterium animalis* (Fig. 5).

It can be seen from Fig. 5(A) and (B) that with the increase in concentration, the antioxidant activity and reducibility of plantain straw hemicellulose B and XOS increased. When the concentration reached 1.6 mg/mL, its activity did not increase significantly. In general, the activity of XOS is higher than that of hemicellulose B, which indicates that the enzyme is responsible for the release of reducing hemiacetal hydroxyl [45]. Compared with previous studies, XOS from plantain straw and *Caulerpa lentillifera* have similar antioxidant activity [46]. Therefore, plantain straw with a wide range of sources may become a more popular source of XOS. Investigating the growth–promoting effect of XOS from plantain straw on *Lactobacillus acidophilus* and *Bifidobacterium animalis* was the focus of the research. It can be seen from Fig. 5(C) that XOS and glucose play a significant role in promoting production during the initial stage of strain growth. During the entire fermentation stage, XOS from plantain straw exhibited a more significant growth–promoting effect compared to glucose. According to the results of the study on the effect of XOS on the growth of *Bifidobacterium animalis in vitro* (Fig. 5(D)), XOS exhibited a more favorable probiotic effect. Therefore, plantain straw XOS has a probiotic–promoting effect on *Lactobacillus acidophilu* and *Bifidobacterium animalis* [47]. Compared with the previous research [48], plantain XOS has a more stable and significant growth–promoting effect.

## 4 Conclusion

To improve the utilization of plantain straw, hemicellulose B was extracted from it. Hemicellulose B was used with the Sevag method to remove proteins and nucleic acids. Ultraviolet–visible scanning showed that the proteins and nucleic acids had been completely removed. Hemicellulose B was hydrolyzed to obtain monosaccharides, which were determined by HPLC to be composed of 13 monosaccharides such as L– guluronic acid. Among them, the contents of xylose, galacturonic acid, arabinose, glucose and rhamnose are relatively high. XOS were obtained through the enzymatic hydrolysis of hemicellulose B using xylanase. SEM observation showed that the surface of XOS was crosslinked and rough. FT–IR analysis revealed that the oligosaccharides contained a β–glycosidic bond. NMR analysis revealed that XOS contain methyl and methoxy groups and are connected by 1–4 glycosides bonds. In addition, compared to hemicellulose B, XOS from plantain straw exhibited superior DPPH scavenging ability and higher reducibility. The analysis of antibacterial activity showed that plantain oligosaccharide had beneficial probiotic effects on *Lactobacillus acidophilus* and *Bifidobacterium animalis.* As a result, XOS from plantain straw has promising applications as additives in food and feed. Additionally, it holds significant research potential in various other fields such as beauty and medicine.

## CRediT authorship contribution statement

**Wenming Jiang:** Writing – original draft. **Fang Li:** Validation.

## Data availability statement

All data generated or analyzed during this study are included in this published article.

## Declaration of competing interest

The authors declare that they have no known competing financial interests or personal relationships that could have appeared to influence the work reported in this paper.
